# Effect of tolvaptan in Japanese patients with autosomal dominant polycystic kidney disease: a post hoc analysis of TEMPO 3:4 and TEMPO Extension Japan

**DOI:** 10.1007/s10157-021-02083-y

**Published:** 2021-06-04

**Authors:** Satoru Muto, Tadashi Okada, Yoshiyuki Shibasaki, Tatsuki Ibuki, Shigeo Horie

**Affiliations:** 1 Department of Advanced Informatics for Genetic Disease, Juntendo University Graduate School of Medicine, Hongo 2-1-1, Bunkyo-ku, Tokyo, 113-8421 Japan; 2 Department of Urology, Juntendo University Graduate School of Medicine, Hongo 2-1-1, Bunkyo-ku, Tokyo, 113-8421 Japan; 3 Department of Clinical Development, Otsuka Pharmaceutical Co., Ltd., Osaka, Japan; 4 Medical Affairs, Otsuka Pharmaceutical Co., Ltd., Tokyo, Japan

**Keywords:** Tolvaptan, Autosomal dominant polycystic kidney disease (ADPKD), Estimated glomerular filtration rate (eGFR), Total kidney volume (TKV), TEMPO 3:4, TEMPO Extension Japan (TEMPO-EXTJ)

## Abstract

**Background:**

Autosomal dominant polycystic kidney disease (ADPKD) is a progressive condition that eventually leads to end-stage renal disease. A phase 3 trial of tolvaptan (TEMPO 3:4; NCT00428948) and its open-label extension (TEMPO Extension Japan: TEMPO-EXTJ; NCT01280721) were conducted in patients with ADPKD. In this post hoc analysis, effects on renal function and the safety profile of tolvaptan were assessed over a long-term period that included the 3-year TEMPO 3:4 and the approximately 3-year TEMPO-EXTJ trials.

**Methods:**

Patients from Japanese trial sites who completed TEMPO 3:4 were offered participation in TEMPO-EXTJ. Patients whose efficacy parameters were measured at year 2 in TEMPO-EXTJ for efficacy evaluation were included. The annual slope of the estimated glomerular filtration rate (eGFR) and growth in total kidney volume (TKV) were analyzed.

**Results:**

In patients who received tolvaptan in TEMPO 3:4 and TEMPO-EXTJ, the annual slope of eGFR (mL/min/1.73 m^2^) was − 3.480 in TEMPO 3:4 and − 3.417 in TEMPO-EXTJ, with no apparent effect of an approximately 3.6-month off-treatment interval between the two trials. In patients who received a placebo in TEMPO 3:4 before initiating tolvaptan in TEMPO-EXTJ, the slope of eGFR was significantly less steep from TEMPO 3:4 (− 4.287) to TEMPO-EXTJ (− 3.364), a difference of 0.923 (*P* = 0.0441).

**Conclusion:**

The TEMPO-EXTJ trial supports a sustained beneficial effect of tolvaptan on eGFR. In patients who received a placebo in TEMPO 3:4, initiation of tolvaptan in TEMPO-EXTJ was associated with a significant slowing of eGFR decline.

**Supplementary Information:**

The online version contains supplementary material available at 10.1007/s10157-021-02083-y.

## Introduction

Autosomal dominant polycystic kidney disease (ADPKD) is an inherited disease characterized by the development of renal cysts leading to progressive kidney enlargement and irreversible decline in kidney function, inexorably resulting in end-stage renal disease [1, 2]. Although ADPKD is considered a slowly progressing disease, once kidney volume reaches a critical size, the glomerular filtration rate (GFR) undergoes a sharp decline [3]. In 2012, the primary efficacy analysis of the worldwide, pivotal, phase 3 Tolvaptan Efficacy and Safety in Management of Autosomal Dominant Polycystic Kidney Disease and Its Outcomes 3:4 (TEMPO 3:4) trial demonstrated that tolvaptan, a non-peptide antagonist of the human arginine vasopressin V2-receptor, slowed the rate of total kidney volume (TKV) growth [4]. A subgroup analysis of the Japanese patients from the TEMPO 3:4 trial confirmed the benefits of tolvaptan in this population [5]. In 2014, Japan was the first country to approve tolvaptan for the treatment of patients with ADPKD, based on evidence that it slows TKV growth and renal function decline over a 3-year period.

Apart from ADPKD, tolvaptan has been approved in Japan for use in heart failure and liver cirrhosis patients with fluid volume overload. Daily doses in these indications are 7.5 to 15 mg, and the duration of treatment is several weeks [6, 7]. For patients with ADPKD, tolvaptan is administered for a longer duration and at higher daily doses (60 to 120 mg) than other diseases in order to suppress the V2 receptor for 24 h a day. Furthermore, it was found that, on unblinding of the TEMPO 3:4 trial, an imbalance in serum alanine aminotransferase (s-ALT) elevations was observed (4.9% vs. 1.2%) in patients on tolvaptan [4]. Therefore, long-term safety data on the use of tolvaptan in the treatment of patients with ADPKD is needed. The open-label TEMPO Extension Japan (TEMPO-EXTJ) trial was designed to provide additional years of data on long-term safety and efficacy of tolvaptan in patients who completed the TEMPO 3:4 trial in Japan; safety results from the extension have been published [8].

The present post hoc analysis examined the effects of tolvaptan in Japanese patients with ADPKD during the TEMPO 3:4 and TEMPO-EXTJ trials. Variables assessed included effects on TKV growth and decline in renal function, as well safety and tolerability.

## Methods

### Study design

#### TEMPO 3:4 trial

The designs of TEMPO 3:4 trial and of the subgroup analysis of Japanese participants have been published [4, 5]. Briefly, eligible Japanese patients were 20–50 years of age, with a diagnosis of ADPKD according to the Ravine criteria, TKV ≥ 750 mL on MRI, and creatinine clearance ≥ 60 mL/min. Patients were randomly assigned in a 2:1 ratio to receive tolvaptan or placebo. Daily doses of study drugs were titrated according to tolerability from 60 to 120 mg/day. The treatment period was 36 months.

#### TEMPO-EXTJ trial

The trial design has been published [8]. In brief, eligibility criteria included: completion of the 3-year treatment period in TEMPO 3:4 trial (tolvaptan or placebo), willingness to continue treatment, and resolution of any adverse events experienced during the TEMPO 3:4 trial. Patients were excluded if they had a low estimated GFR (eGFR), i.e., < 15 mL/min/1.73 m^2^. All patients received tolvaptan in this study.

### Patients

Patients from Japanese trial sites who completed the TEMPO 3:4 trial were offered the opportunity to participate in the TEMPO-EXTJ trial. Patients whose TKV or eGFR were measured at year 2 in the TEMPO-EXTJ trial for efficacy evaluation were included. The reason why efficacy was evaluated at year 2 was the small sample size in patients whose efficacy parameters could be evaluated after year 2 in the TEMPO-EXTJ trial. Patients who received tolvaptan, even once, throughout the TEMPO-EXTJ trial for safety evaluation were included.

### Ethical conduct

The trials were conducted in accordance with the ethical principles originating from the Declaration of Helsinki and in compliance with good clinical practice guidelines. The protocols were approved by the institutional review board at each trial site. Written informed consent was obtained from all participating patients. Both trials were registered with ClinicalTrials.gov (TEMPO 3:4 trial, NCT00428948; TEMPO-EXTJ trial, NCT01280721).

### Evaluations

**Fig. 1:**
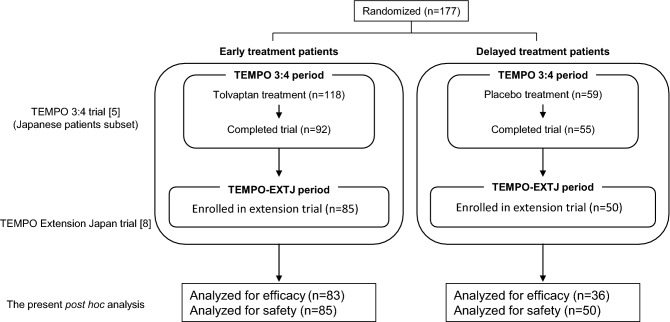
Patient flow. Early treatment and delayed treatment patients are defined by allocation to the tolvaptan or placebo treatment arms in the TEMPO 3:4 trial. The overall duration of follow-up is divided into the TEMPO 3:4 trial and the TEMPO-EXTJ trial periods. Patients analyzed for efficacy were defined as those whose efficacy parameters were measured during the 2 years in the TEMPO-EXTJ trial. Safety was evaluated for patients with receiving tolvaptan, even once, throughout the TEMPO-EXTJ trial

As shown in Fig. 1, patients were categorized based on their treatment arm in the TEMPO 3:4 trial, either as early treatment patients (received tolvaptan in the TEMPO 3:4 trial) or delayed treatment patients (received placebo in the TEMPO 3:4 trial). The overall duration of follow-up in this post hoc analysis was divided into the TEMPO 3:4 trial period and the TEMPO-EXTJ trial period. Patient assessments conducted during an intervening off-treatment period, which consisted of the observation period after the TEMPO 3:4 trial and the screening period at the start of the TEMPO-EXTJ trial, were not included in this analysis.

The slope of eGFR from baseline and percent changes in TKV from baseline were calculated. Baseline eGFR was defined as the end of titration in the TEMPO 3:4 trial or the TEMPO-EXTJ trial. Data on eGFR were collected during clinical laboratory assessments, and they are expressed as mean values using the Chronic Kidney Disease Epidemiology Collaboration Equation as modified for Japanese patients: eGFR (mL/min/1.73 m^2^) = 194 × serum creatinine^−1.094^ × age^−0.287^ (for males), and 194 × serum creatinine^−1.094^ × age^−0.287^ × 0.739 (for females) [9]. Data on TKV were measured at baseline and every 12 months after initiation of study treatment in each trial.

Adverse event data were collected throughout each trial. Hepatic adverse events observed in the TEMPO-EXTJ trial have already been reported [8].

### Statistical analysis

The slope of eGFR was calculated by regressing renal function (starting at the end of titration) against time by patient, with time defined as (observation date − end of titration date + 1)/365.25. Differences in the slopes of eGFR decline between the TEMPO 3:4 trial and TEMPO-EXTJ trial periods were evaluated by the Wald type test using Mixed-Effect Model Repeated Measures with random effects of intercept and time with unknown variance–covariance structure.

The percentage change of TKV from the baseline of the TEMPO 3:4 trial was calculated. Comparisons of adverse events between the TEMPO 3:4 trial and TEMPO-EXTJ trial periods were performed using the McNemar test. *P* values and 95% confidence intervals (CIs) were calculated, with statistical significance defined as *P* < 0.05. All statistical analyses were performed using SAS 9.4 (SAS Institute, Cary, North Carolina, USA).

## Results

### Patients

In the Japanese subpopulation of the TEMPO 3:4 trial, 118 patients were in the tolvaptan arm, and 59 were in the placebo arm (Fig. 1), of whom 92 patients in the tolvaptan arm and 55 patients in the placebo arm completed the trial (total: 147 patients). Of the 147 completers, 135 patients entered the TEMPO-EXTJ trial, including 85 who received tolvaptan in the TEMPO 3:4 trial (early treatment patients) and 50 who received placebo (delayed treatment patients). Tolvaptan was administered throughout the TEMPO 3:4 and TEMPO-EXTJ trials in the early treatment patients and initiated in the TEMPO-EXTJ trial in the delayed treatment patients.

In the present post hoc analysis for efficacy evaluation, 83 patients in the early treatment and 36 in the delayed treatment groups were included. For the safety evaluation, 85 patients in the early treatment and 50 in the delayed treatment groups were included. Patients who were excluded because it was impossible to evaluate TKV or eGFR in this efficacy analysis were 2 in the early treatment and 14 in the delayed treatment groups.

The demographics and clinical characteristics of the early treatment and delayed treatment patients, based on efficacy evaluation, at the baseline of each trial are shown in Table 1. The parameters relevant to the progression of ADPKD were as follows (reported as the TEMPO 3:4 trial period vs. the TEMPO-EXTJ trial period); (1) TKV in early treatment patients: 1493 ± 600 vs. 1709 ± 742 (mL); TKV in delayed treatment patients: 1538 ± 568 vs. 1802 ± 685 (mL); (2) serum creatinine in early treatment patients: 0.99 ± 0.31 vs. 1.09 ± 0.42 (mg/dL); serum creatinine in delayed treatment patients: 0.94 ± 0.23 vs. 1.15 ± 0.42 (mg/dL); (3) eGFR in early treatment patients: 64.8 ± 17.0 vs. 59.9 ± 20.6 (mL/min/1.73 m^2^); eGFR in delayed treatment patients: 67.3 ± 15.4 vs. 54.9 ± 17.8 (mL/min/1.73 m^2^). Thus, the average TKV was smaller and the average eGFR was higher in early treatment patients vs. delayed treatment patients at baseline of the TEMPO-EXTJ trial.

**Table 1:** Demographics and clinical characteristics of patients in the early treatment and delayed treatment groups at baseline in each trial based on efficacy evaluation

	TEMPO 3:4 trial		TEMPO-EXTJ trial	
	Early treatment patients (Tolvaptan) (*n* = 83)	Delayed treatment patients (Placebo) (*n* = 36)	Early treatment patients (Tolvaptan) (*n* = 83)	Delayed treatment patients (Tolvaptan) (*n* = 36)
Demographic characteristics				
Sex (male)	44 (53.0)	21 (58.3)	44 (53.0)	21 (58.3)
Age (years)	39 ± 6	41 ± 6	42 ± 6	44 ± 6
Height (cm)	167.3 ± 8.8	169.4 ± 6.5	167.4 ± 8.9	169.3 ± 6.5
Weight (kg)	66.2 ± 12.9	65.4 ± 12.0	67.7 ± 13.9	66.1 ± 12.6
Average dosage of tolvaptan	95.6 ± 22.7	N/A	93.7 ± 24.3	87.5 ± 23.5
Current medication				
Angiotensin converting enzyme (ACE) inhibitor	4 (4.8)	6 (16.7)	5 (6.0)	2 (5.6)
Angiotensin II receptor blocker (ARB)	45 (54.2)	22 (61.1)	45 (54.2)	19 (52.8)
ACE inhibitor, ARB, or both	47 (56.6)	23 (63.9)	48 (57.8)	20 (55.6)
Calcium-channel blocker	26 (31.3)	12 (33.3)	26 (31.3)	11 (30.6)
Polycystic kidney disease characteristics				
Blood pressure (mm Hg)				
Systolic	125.2 ± 13.7	127.0 ± 13.5	126.2 ± 12.9	123.9 ± 10.4
Diastolic	80.8 ± 13.5	83.1 ± 9.0	81.1 ± 8.8	81.9 ± 8.4
Total kidney volume (TKV) (mL)	1493 ± 600	1538 ± 568	1709 ± 742	1802 ± 685
Serum creatinine (mg/dL)	0.99 ± 0.31	0.94 ± 0.23	1.09 ± 0.42	1.15 ± 0.42
Estimated GFR (mL/min/1.73m^2^) Japanese	64.8 ± 17.0	67.3 ± 15.4	59.9 ± 20.6	54.9 ± 17.8

*TEMPO* Tolvaptan Efficacy and Safety in Management of Autosomal Dominant Polycystic Kidney Disease and Its Outcomes, *GFR* glomerular filtration rate

Data are expressed as mean ± standard deviation or n (percentage)

Estimated GFR was calculated by the Chronic Kidney Disease Epidemiology Collaboration Equation modified for Japanese patients

Discontinuation period was excluded

### Treatment

In early treatment patients, average daily doses of tolvaptan during the TEMPO 3:4 and TEMPO-EXTJ trials were 95.64 ± 22.72 (mg/day) and 92.85 ± 24.69 (mg/day), respectively. In delayed treatment patients, the average daily dose of tolvaptan during the TEMPO-EXTJ trial was 83.81 ± 24.44 (mg/day). The time interval (expressed as mean/median) between completion of the TEMPO 3:4 trial and the initiation of the TEMPO-EXTJ trial was 3.6/3.3 months for early treatment patients and 3.7/3.5 months for delayed treatment patients.

### Slope of eGFR

**Fig. 2:**
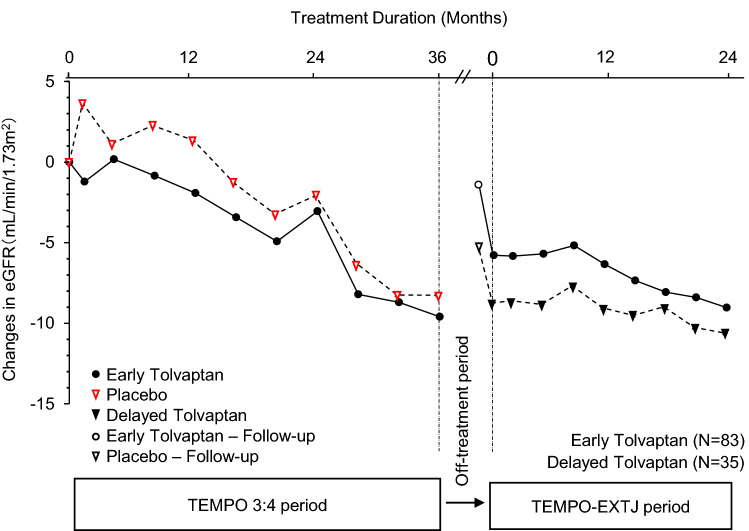
Changes in eGFR over time from baseline of the TEMPO 3:4 trial. Changes in eGFR over time from the TEMPO 3:4 trial baseline to Month 24 of the TEMPO-EXTJ trial in early treatment patients and delayed treatment patients. The off-treatment interval includes the follow-up period after the TEMPO 3:4 trial and the screening period for the TEMPO-EXTJ trial. There is one missing data point of baseline eGFR in the placebo treatment group

In 36 delayed treatment patients, eGFR was not measured in one patient, and 35 patients were included in evaluation of eGFR. Changes in eGFR over time from the baseline of the TEMPO 3:4 trial through the TEMPO-EXTJ trial are shown in Figure 2. The annual slope of eGFR in each period is shown by the treatment group in Table 2. In the delayed treatment patients, the annual slopes in the TEMPO 3:4 trial and TEMPO-EXTJ trial periods were − 4.287 and − 3.364 (mL/min/1.73 m^2^), respectively, and the difference was statistically significant (0.923; 95% CI, 0.024 to 1.821; *P* = 0.0441). In early treatment patients, in contrast, annual slopes in the TEMPO 3:4 trial and the TEMPO-EXTJ trial periods were − 3.480 and − 3.417 (mL/min/1.73 m^2^), and no statistically significant difference was observed (0.064; 95% CI, − 0.564 to 0.691; *P* = 0.8425). The data for the early treatment group indicate that the effects of tolvaptan on eGFR were not affected by the off-treatment period.

**Table 2:** Decline in renal function (estimated GFR)

Slope comparison	Period	Treatment	Slope (mL/min/1.73 m^2^)	Difference	95% CI	*P* value
Delayed treatment patients	TEMPO 3:4 trial	Placebo	− 4.287	0.923	0.024 to 1.821	0.0441
	TEMPO-EXTJ trial	Tolvaptan	− 3.364			
Early treatment patients	TEMPO 3:4 trial	Tolvaptan	− 3.480	0.064	− 0.564 to 0.691	0.8425
	TEMPO-EXTJ trial	Tolvaptan	− 3.417			

*CI* confidence interval, *GFR* glomerular filtration rate, *TEMPO* Tolvaptan Efficacy and Safety in Management of Autosomal Dominant Polycystic Kidney Disease and Its Outcomes

Estimated GFR was calculated by the Chronic Kidney Disease Epidemiology Collaboration Equation modified for Japanese patients

Summary statistics are based on the slope of change, obtained by regressing renal function (starting at end of titration) against time by patient. The time variable used in the regression was (observation date − end of titration date + 1)/365.25

### Percentage change from baseline in TKV growth

**Fig. 3:**
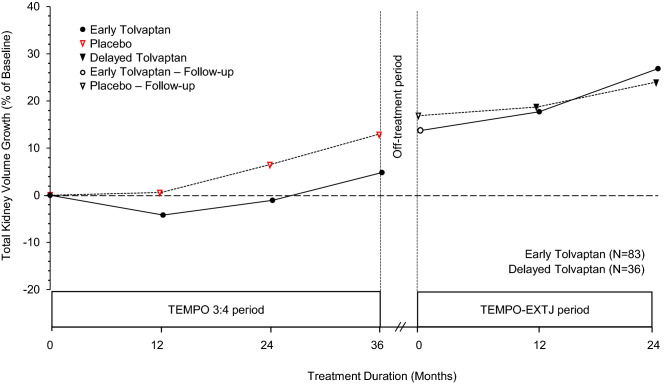
Percentage changes in TKV over time from baseline of the TEMPO 3:4 trial. Percentage changes in TKV from the TEMPO 3:4 trial baseline to Month 24 of the TEMPO-EXTJ trial in early treatment patients and delayed treatment patients. The off-treatment interval includes the follow-up period after the TEMPO 3:4 trial and the screening period for the TEMPO-EXTJ trial

Figure 3 shows the growth of TKV overtime in early treatment and delayed treatment patients expressed as percentage change from the TEMPO 3:4 trial baseline. In the delayed treatment patients, percentage changes in TKV from baseline were 0.6%, 6.6%, and 13.0% at 12, 24, and 36 months, respectively, of the TEMPO 3:4 trial period and 19.0% and 24.2% at 12 and 24 months, respectively, of the TEMPO-EXTJ trial period. The early treatment patients showed percentage changes in TKV from baseline of − 4.2%, − 1.1%, and 4.8% at 12, 24, and 36 months, respectively, of the TEMPO 3:4 trial period, and 18.0% and 27.0% at 12 and 24 months, respectively, of the TEMPO-EXTJ trial period. TKV growth (percent change from baseline) in the first year of the TEMPO-EXTJ trial period was 3.9% in early treatment patients and 1.8% in delayed treatment patients.

### Adverse events

The demographics and clinical characteristics of the early treatment and delayed treatment patients, based on safety evaluation, at baseline of each trial are shown in Supplement 1. Adverse events that occurred throughout the 2 trial periods are shown in Supplement 2. During the TEMPO-EXTJ trial period, the incidence of aquaretic adverse events (AAEs) significantly increased in delayed treatment patients as they initiated tolvaptan during the extension trial, compared to the incidence in this group during the TEMPO 3:4 trial period [8].

## Discussion

The TEMPO 3:4 trial enrolled patients with earlier stage ADPKD (estimated creatinine clearance, ≥ 60 mL/min) and tolvaptan reduced rates of kidney growth and decline in eGFR [4]. We previously reported the long-term safety profile of tolvaptan in the TEMPO-EXTJ trial [8]. In the present post hoc analysis, the long-term efficacy and safety of tolvaptan were investigated throughout two trials, TEMPO 3:4 and TEMPO-EXTJ trials, the latter of which enrolled Japanese participants who had completed the TEMPO 3:4 trial and started open-label tolvaptan treatment. In the TEMPO 4:4 trial, an open-label, global extension trial of the TEMPO 3:4 trial, no Japanese patients, in fact, no Asian patients, were included; therefore, no efficacy information was reported for them. The present post hoc analysis examined the effects of tolvaptan on TKV growth and decline in renal function in Japanese patients with ADPKD during the TEMPO 3:4 and TEMPO-EXTJ trials. In this analysis of renal function, early treated patients maintained the treatment benefit of tolvaptan for renal function in the TEMPO 3:4 trial for additional years, with no evident reduction in benefit following the off-treatment interval between trials. This result was similar to that observed in the TEMPO 4:4 trial [10]. In delayed treatment patients, eGFR decline in the TEMPO-EXTJ trial was slower than in the TEMPO 3:4 trial, even though average baseline eGFR values in the TEMPO-EXTJ trial were lower than baseline eGFR values in the TEMPO 3:4 trial. The slopes of eGFR in the TEMPO-EXTJ trial were similar in early treatment and delayed treatment patients (Table 2) and similar to those in the TEMPO 4:4 trial [10]. In the recent REPRISE trial (NCT02160145), a randomized placebo-controlled trial of tolvaptan in patients with later-stage ADPKD (18–55 years of age: eGFR 25–65 mL/min/1.73 m^2^ or 56–65 years of age, 25–44 mL/min/1.73 m^2^) compared with patients in the TEMPO 3:4 trial, tolvaptan was associated with a slower decline in eGFR than placebo [11]. Taken together, these findings show that tolvaptan slows deterioration in renal function of ADPKD patients at advanced CKD stages, as well as earlier CKD stages, with efficacy maintained at least for 5 years of tolvaptan use.

It is difficult to evaluate tolvaptan efficacy in slowing TKV growth during the TEMPO-EXTJ trial, given that this extension trial was open-label and did not have a placebo group. In the TEMPO 4:4 trial, extrapolation of the placebo-treated group’s trajectory beyond 3 years suggested that TKV growth would be approximately 40% after 5 years, whereas that the observed rate of TKV growth in both the early tolvaptan treatment and delayed tolvaptan groups was approximately 30% over 5 years [10]. Extrapolating data from the present analysis in a similar fashion yield projected TKV growth of approximately 33% over 5 years in placebo-treated patients; the actual rates of TKV growth in early treatment and delayed treatment patients were approximately 27% and 24%, respectively (Fig. 3). Comparison of the projected and actual values suggests that both early treatment and delayed treatment patients obtained TKV benefits (i.e., slowing in rate of TKV growth) from tolvaptan during the TEMPO-EXTJ trial. In the delayed treatment patients of the TEMPO-EXTJ trial, the largest effect of tolvaptan on TKV growth in the first year, similar to the TEMPO 4:4 trial, was not clearly observed. It is not clear whether the reason for this difference is due to intrinsic or extrinsic factors, such as ethnic diversity. However, as in the TEMPO 4:4 trial, the effects of tolvaptan on TKV growth in delayed treatment patients were relatively larger than that in early treatment patients in the TEMPO-EXTJ trial. Torres et al. concluded that the effect of tolvaptan on TKV growth in the TEMPO 3:4 trial was due to the following two mechanisms: the first one was relatively large due to the antisecretory effect of tolvaptan observed in the first year after starting treatment, and a relatively small reduction in cyst volume was observed throughout the administration period [10]. In the present post hoc analysis, percentage changes in TKV from baseline in the first year of the TEMPO-EXTJ trial period were 1.8 and 3.9% in the delayed and early treatment patients, respectively. Therefore, it was considered that the effects of tolvaptan on TKV growth in delayed treatment patients receiving tolvaptan for the first time in the TEMPO-EXTJ trial were greater than in early treatment patients due to the antisecretory effect.

Safety data from the TEMPO-EXTJ trial have already been published [8]. The most common adverse events were AAEs such as thirst, pollakiuria, and polyuria; these adverse events are not unexpected, since they are associated with the pharmacological action of tolvaptan. In early treatment patients, the incidence of thirst was lower in the TEMPO-EXTJ trial period than in the TEMPO 3:4 trial period (*P* = 0.0455), likely because treated patients in the TEMPO 3:4 trial adjusted to this event (Supplement 2).

A limitation of this analysis is the absence of a long-term placebo control arm because the TEMPO-EXTJ trial was an open-label extension; thus, comparative analysis between long-term tolvaptan treatment and an untreated ADPKD population was not possible. Comparison of the early treatment and delayed treatment patients is further complicated by the unequal distribution of dropouts in the prior trial and the option to accept or decline participation in the extension trial. The extension continued until regulatory approval of tolvaptan in Japan; therefore, the treatment periods varied widely. Furthermore, due to the slow progression of ADPKD, it is unknown whether the length of follow-up is sufficient to evaluate long-term efficacy. For these reasons, future trials should collect follow-up data over long periods.

In conclusion, the TEMPO 3:4 and TEMPO-EXTJ trials showed the sustained beneficial effect of tolvaptan on eGFR decline in Japanese ADPKD patients over 5 years. The off-treatment interval between the 2 trials did not impact the efficacy of tolvaptan in the early treatment patients. It is considered that the early treatment patients received a greater therapeutic benefit from tolvaptan than delayed treatment patients during the two trials. In the delayed treatment patients, the slope of eGFR decline in the TEMPO-EXTJ trial period decreased relative to the TEMPO 3:4 trial period, indicating treatment benefit following tolvaptan initiation, and as would be expected, AAEs related to the pharmacological mechanism of action increased significantly in the TEMPO-EXTJ trial period compared to the TEMPO 3:4 trial period.

## Supplementary Information

Below is the link to the electronic supplementary material.

Supplementary file1 (DOCX 41 kb)

Supplementary file2 (DOCX 36 kb)

## References

[1] Torres VE, Harris PC, Pirson Y (2007). Autosomal dominant polycystic kidney disease. Lancet.

[2] Grantham JJ (2008). Clinical practice. Autosomal dominant polycystic kidney disease. N Engl J Med.

[3] Grantham JJ (2015). Rationale for early treatment of polycystic kidney disease. Pediatr Nephrol.

[4] Torres VE, Chapman AB, Devuyst O (2012). Tolvaptan in patients with autosomal dominant polycystic kidney disease. N Engl J Med.

[5] Muto S, Kawano H, Higashihara E (2015). The effect of tolvaptan on autosomal dominant polycystic kidney disease patients: a subgroup analysis of the Japanese patient subset from TEMPO 3:4 trial. Clin Exp Nephrol.

[6] Matsuzaki M, Hori M, Izumi T, for the Tolvaptan Investigators (2011). Efficacy and safety of tolvaptan in heart failure patients with volume overload despite the standard treatment with conventional diuretics: a phase III, randomized, double-blind, placebo-controlled Study (QUEST Study). Cardiovasc Drugs Ther.

[7] Sakaida I, Kawazoe S, Kajimura K (2014). Tolvaptan for improvement of hepatic edema: a phase 3, multicenter, randomized, double-blind, placebo-controlled trial. Hepatol Res.

[8] Muto S, Okada T, Yasuda M (2017). Long-term safety profile of tolvaptan in autosomal dominant polycystic kidney disease patients: TEMPO Extension Japan trial. Drug Healthc Patient Saf.

[9] Matsuo S, Imai E, Horio M (2009). Revised equations for estimated GFR from serum creatinine in Japan. Am J Kidney Dis.

[10] Torres VE, Chapman AB, Devuyst O (2018). Multicenter, open-label, extension trial to evaluate the long-term efficacy and safety of early versus delayed treatment with tolvaptan in autosomal dominant polycystic kidney disease: the TEMPO 4:4 Trial. Nephrol Dial Transplant.

[11] Torres VE, Chapman AB, Devuyst O, REPRISE Trial Investigators (2017). Tolvaptan in later-stage autosomal dominant polycystic kidney disease. N Engl J Med.

