# Supplementary material for: Effect of tolvaptan in Japanese patients with autosomal dominant polycystic kidney disease: a post hoc analysis of TEMPO 3:4 and TEMPO Extension Japan
Source: Clin Exp Nephrol. 2021 Jun 4;25(9):1003–10. doi: 10.1007/s10157-021-02083-y (PMC8357671; doi:10.1007/s10157-021-02083-y)
Supplement: Supplementary file 1 — Supplementary file1 (DOCX 41 kb) [file 10157_2021_2083_MOESM1_ESM.docx]

| **Supplement 2. Adverse events** |  |  |  |  |  |  |
| --- | --- | --- | --- | --- | --- | --- |
| Events（SOC＼PT） | Early treatment patients (n = 85) | | | Delayed treatment patients (n = 50) | | |
|  | TEMPO 3:4 trial | TEMPO-EXTJ trial | *P* value ^a)^ | TEMPO 3:4 trial | TEMPO-EXTJ trial | *P* value ^a)^ |
| Treatment | Tolvaptan | Tolvaptan |  | Placebo | Tolvaptan |  |
| Cardiac disorders |  |  |  |  |  |  |
| Palpitations | 9 (10.6) | 3 (3.5) | 0.0339 | 0 (0.0) | 0 (0.0) | ‐ |
| Gastrointestinal disorders |  |  |  |  |  |  |
| Abdominal discomfort | 3 (3.5) | 3 (3.5) | NS | 2 (4.0) | 5 (10.0) | NS |
| Abdominal pain | 8 (9.4) | 3 (3.5) | NS | 5 (10.0) | 3 (6.0) | NS |
| Abdominal pain upper | 12 (14.1) | 3 (3.5) | 0.0027 | 8 (16.0) | 2 (4.0) | NS |
| Constipation | 14 (16.5) | 5 (5.9) | 0.0201 | 2 (4.0) | 2 (4.0) | NS |
| Dental caries | 2 (2.4) | 4 (4.7) | NS | 3 (6.0) | 6 (12.0) | NS |
| Diarrhea | 14 (16.5) | 13 (15.3) | NS | 8 (16.0) | 8 (16.0) | NS |
| Gastroesophageal reflux disease | 9 (10.6) | 4 (4.7) | NS | 4 (8.0) | 1 (2.0) | NS |
| Nausea | 13 (15.3) | 5 (5.9) | 0.0325 | 4 (8.0) | 6 (12.0) | NS |
| Toothache | 1 (1.2) | 1 (1.2) | NS | 5 (10.0) | 1 (2.0) | NS |
| Vomiting | 11 (12.9) | 7 (8.2) | NS | 2 (4.0) | 3 (6.0) | NS |
| General disorders and administration site conditions |  |  |  |  |  |  |
| Edema peripheral | 8 (9.4) | 4 (4.7) | NS | 5 (10.0) | 4 (8.0) | NS |
| Pyrexia | 8 (9.4) | 6 (7.1) | NS | 8 (16.0) | 2 (4.0) | 0.0339 |
| Thirst | 78 (91.8) | 70 (82.4) | 0.0455 | 15 (30.0) | 34 (68.0) | 0.0001 |
| Hepatobiliary disorders |  |  |  |  |  |  |
| Hepatic function abnormal | 6 (7.1) | 2 (2.4) | NS | 2 (4.0) | 12 (24.0) | 0.0039 |
| Infections and infestations |  |  |  |  |  |  |
| Cystitis | 2 (2.4) | 2 (2.4) | NS | 5 (10.0) | 0 (0.0) | ‐ |
| Gastroenteritis | 4 (4.7) | 9 (10.6) | NS | 5 (10.0) | 4 (8.0) | NS |
| Influenza | 7 (8.2) | 13 (15.3) | NS | 6 (12.0) | 7 (14.0) | NS |
| Nasopharyngitis | 61 (71.8) | 62 (72.9) | NS | 39 (78.0) | 32 (64.0) | NS |
| Pharyngitis | 2 (2.4) | 3 (3.5) | NS | 5 (10.0) | 1 (2.0) | NS |
| Investigations |  |  |  |  |  |  |
| Blood creatinine increased | 8 (9.4) | 7 (8.2) | NS | 3 (6.0) | 5 (10.0) | NS |
| Metabolism and nutrition disorders |  |  |  |  |  |  |
| Hyperuricemia | 13 (15.3) | 20 (23.5) | NS | 3 (6.0) | 9 (18.0) | NS |
| Musculoskeletal and connective tissue disorders |  |  |  |  |  |  |
| Back pain | 24 (28.2) | 19 (22.4) | NS | 15 (30.0) | 11 (22.0) | NS |
| Myalgia | 9 (10.6) | 1 (1.2) | 0.0114 | 4 (8.0) | 1 (2.0) | NS |
| Nervous system disorders |  |  |  |  |  |  |
| Dizziness | 14 (16.5) | 7 (8.2) | NS | 8 (16.0) | 3 (6.0) | NS |
| Headache | 28 (32.9) | 17 (20.0) | 0.0343 | 14 (28.0) | 9 (18.0) | NS |
| Hypoesthesia | 2 (2.4) | 1 (1.2) | NS | 5 (10.0) | 2 (4.0) | NS |
| Renal and urinary disorders |  |  |  |  |  |  |
| Hematuria | 6 (7.1) | 4 (4.7) | NS | 5 (10.0) | 2 (4.0) | NS |
| Pollakiuria | 47 (55.3) | 49 (57.6) | NS | 2 (4.0) | 28 (56.0) | <0.0001 |
| Polyuria | 28 (32.9) | 30 (35.3) | NS | 5 (10.0) | 22 (44.0) | 0.0004 |
| Renal pain | 10 (11.8) | 1 (1.2) | 0.0027 | 11 (22.0) | 1 (2.0) | 0.0016 |
| Respiratory, thoracic and mediastinal disorders |  |  |  |  |  |  |
| Cough | 2 (2.4) | 1 (1.2) | NS | 5 (10.0) | 3 (6.0) | NS |
| Upper respiratory tract inflammation | 3 (3.5) | 15 (17.6) | 0.0047 | 1 (2.0) | 6 (12.0) | 0.0253 |
| Oropharyngeal pain | 9 (10.6) | 2 (2.4) | 0.0348 | 6 (12.0) | 1 (2.0) | NS |
| Vascular disorders |  |  |  |  |  |  |
| Hypertension | 23 (27.1) | 13 (15.3) | 0.0412 | 13 (26.0) | 7 (14.0) | NS |
| N (%), number of patients (%). a) Comparisons of adverse events between study periods were performed using the McNemar test.  Abbreviations: PT, preferred term; SOC, system organ class; TEMPO, Tolvaptan Efficacy and Safety in Management of Autosomal Dominant Polycystic Kidney Disease and Its Outcomes | | | | | | |
| TEMPO 3:4 trial: Medical Dictionary for Regulatory Activities, version 14.1 | | | | | | |
| TEMPO-EXTJ trial: Medical Dictionary for Regulatory Activities, version 17.0 | | | | | | |
