# Supplementary material for: Effect of tolvaptan in Japanese patients with autosomal dominant polycystic kidney disease: a post hoc analysis of TEMPO 3:4 and TEMPO Extension Japan
Source: Clin Exp Nephrol. 2021 Jun 4;25(9):1003–10. doi: 10.1007/s10157-021-02083-y (PMC8357671; doi:10.1007/s10157-021-02083-y)
Supplement: Supplementary file 2 — Supplementary file2 (DOCX 36 kb) [file 10157_2021_2083_MOESM2_ESM.docx]

**Supplement 1. Demographics and clinical characteristics of patients in the early treatment and delayed treatment groups at baseline in each trial based on safety evaluation**

|  | TEMPO 3:4 trial | |  | TEMPO-EXTJ trial | |
| --- | --- | --- | --- | --- | --- |
|  | Early treatment  patients  (Tolvaptan)  (n = 85) | Delayed treatment  patients  (Placebo)  (n = 50) |  | Early treatment  patients  (Tolvaptan)  (n = 85) | Delayed treatment  patients  (Tolvaptan)  (n = 50) |
| Demographic characteristics |  |  |  |  |  |
| Sex (male) | 45 (52.9) | 32 (64.0) |  | 45 (52.9) | 32 (64.0) |
| Age (years) | 39 ± 6 | 41 ± 5 |  | 42 ± 6 | 44 ± 5 |
| Height (cm) | 167.3 ± 8.8 | 170.0 ± 6.4 |  | 167.4 ± 8.9 | 169.8 ± 6.3 |
| Weight (kg) | 66.2 ± 12.9 | 66.7 ± 11.7 |  | 67.7 ± 13.8 | 67.4 ± 12.4 |
| Average dosage of tolvaptan | 95.6 ± 22.7 | N/A |  | 92.9 ± 24.7 | 83.8 ± 24.4 |
| Current medication |  |  |  |  |  |
| Angiotensin converting enzyme (ACE) inhibitor | 4 (4.7) | 8 (16.0) |  | 5 (5.9) | 2 (4.0) |
| Angiotensin II receptor blocker (ARB) | 47 (55.3) | 33 (66.0) |  | 47 (55.3) | 26 (52.0) |
| ACE inhibitor, ARB, or both | 49 (57.6) | 35 (70.0) |  | 50 (58.8) | 27 (54.0) |
| Calcium-channel blocker | 27 (31.8) | 22 (44.0) |  | 27 (31.8) | 19 (38.0) |
| Polycystic kidney disease characteristics |  |  |  |  |  |
| Blood pressure (mm Hg) |  |  |  |  |  |
| Systolic | 125.2 ± 13.8 | 125.8 ± 13.2 |  | 126.1 ± 12.8 | 125.2 ± 9.9 |
| Diastolic | 80.7 ± 13.3 | 81.3 ± 10.1 |  | 80.9 ± 8.8 | 82.9 ± 8.3 |
| Total kidney volume (TKV) (mL) | 1487 ± 597 | 1604 ± 643 |  | 1706 ± 738 | 1994 ± 1016 |
| Serum creatinine (mg/dL) | 1.00 ± 0.32 | 0.99 ± 0.27 |  | 1.12 ± 0.50 | 1.30 ± 0.68 |
| Estimated GFR (mL/min/1.73m^2^) Japanese | 65.3 ± 16.3 | 61.1 ± 16.1 |  | 63.5 ± 22.5 | 55.4 ± 20.5 |

Data are expressed as mean ± standard deviation or n (percentage).

Estimated GFR was calculated by the Chronic Kidney Disease Epidemiology Collaboration Equation modified for Japanese patients.

Discontinuation period was excluded.

Abbreviations: TEMPO, Tolvaptan Efficacy and Safety in Management of Autosomal Dominant Polycystic Kidney Disease and Its Outcomes; GFR, glomerular filtration rate
